# Crystal structure of 5-hy­droxy-5-propyl­barbituric acid

**DOI:** 10.1107/S2056989015018769

**Published:** 2015-10-14

**Authors:** Thomas Gelbrich, Ulrich J. Griesser

**Affiliations:** a University of Innsbruck, Institute of Pharmacy, Innrain 52, 6020 Innsbruck, Austria

**Keywords:** barbiturates, crystal structure, hydrogen bonding, isostructurality, topology, *XPac*

## Abstract

Mol­ecules of the title compound, C_7_H_10_N_2_O_4_, systematic name 5-hy­droxy-5-propyl­pyrimidine-2,4,6(1*H*,3*H*,5*H*)-trione, form a hydrogen-bonded framework which is based on three independent hydrogen bonds, N—H⋯O(carbon­yl), N—H⋯O(hy­droxy) and O—H⋯O(carbon­yl). This framework has the topology of the 5-connected **nov** net. Each mol­ecule is linked to five other mol­ecules *via* six hydrogen bonds, and the descriptor of the hydrogen-bonded structure is *F*6_5_[4^4^.6^6^-**nov**]. The crystal packing is isostructural with that of the previously reported 5-hy­droxy-5-ethyl analogue.

## Abstract 2

Mol­ecules of the title compound are linked *via* N—H⋯O(carbon­yl), N—H⋯O(hy­droxy) and O—H⋯O(carbon­yl) bonds into a 5-connected framework.

## Chemical context   

As part of a systematic investigation of solid-state properties of derivatives of barbituric acid (Gelbrich *et al.*, 2015 ▸; Zencirci *et al.*, 2014 ▸; Rossi *et al.*, 2012 ▸), we are studying the polymorphism of a group of 5-monosubstituted barbituric acids. The title compound is an oxidation product of 5-propyl­barbituric acid, formed during a crystallization experiment and the structure is reported herein. The analogous oxidation product of 5-ethyl­barbituric acid was previously reported by Gatehouse & Craven (1971 ▸).
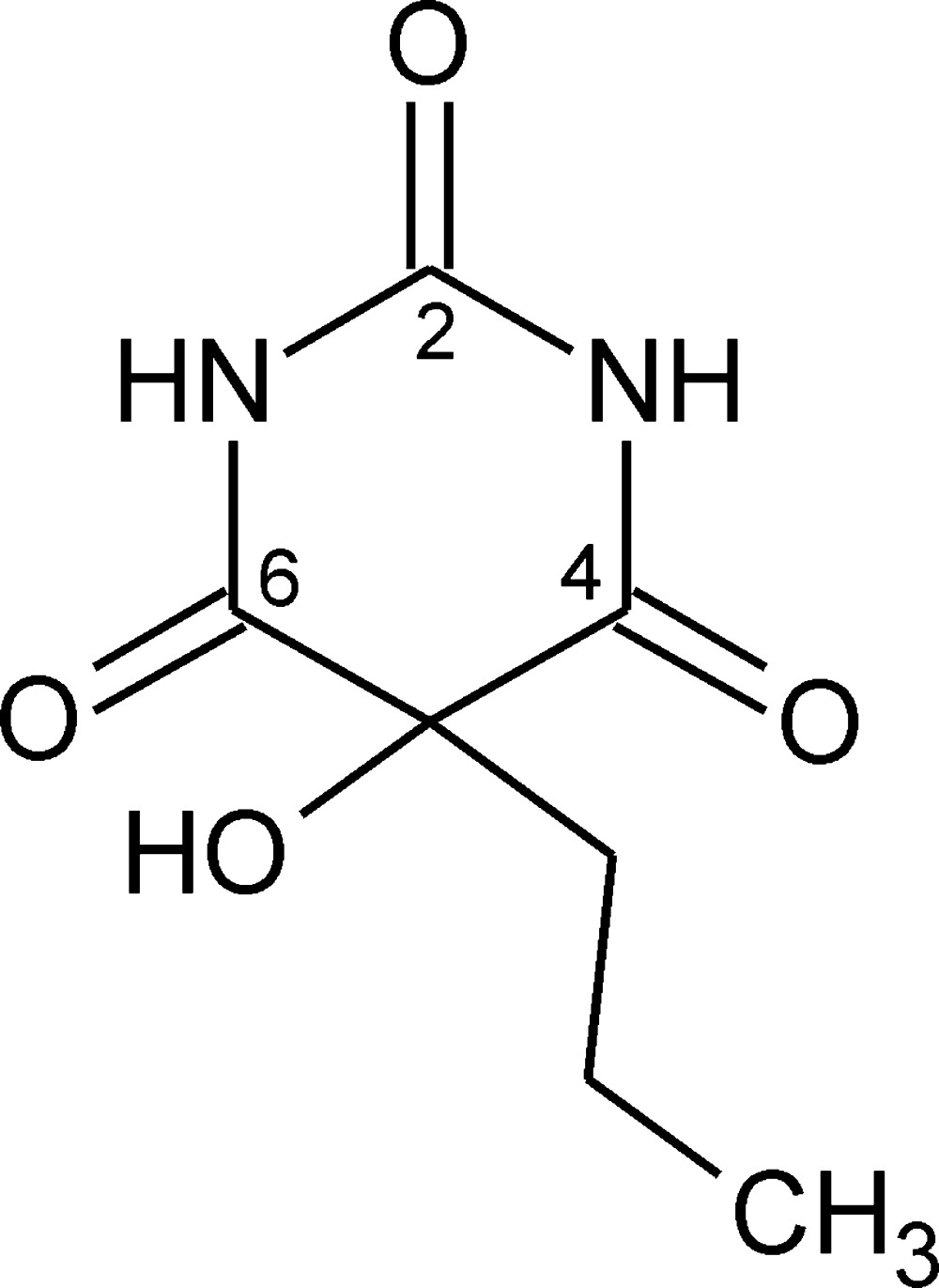


## Structural commentary   

**Figure 1:**
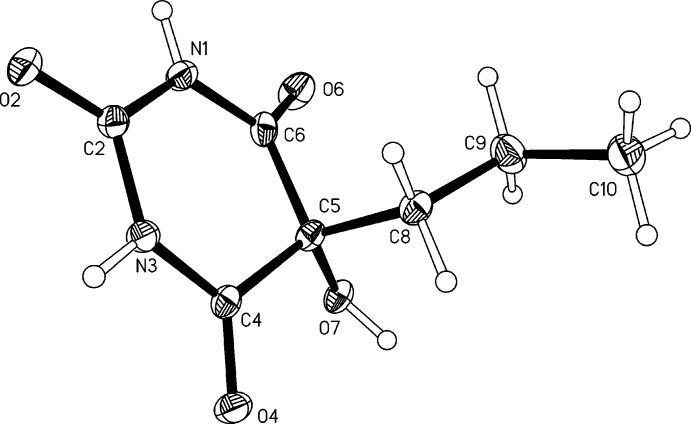
Asymmetric unit with displacement ellipsoids drawn at the 50% probability level and hydrogen atoms drawn as spheres of arbitrary size.

The mol­ecule of the title compound (Fig. 1 ▸) displays a pyrim­idine ring (N1/C2/N3/C4/C5/C6) in a *C*5-envelope conformation. The ring puckering parameters calculated with *PLATON* (Spek, 2009 ▸) are θ = 134.4 (3), Φ = 52.2 (5)° and *Q* = 0.2420 (14) Å. The distance of C5 from the mean plane defined by the other four ring atoms [maximum deviation: N3; −0.033 (1) Å] is −0.342 (2) Å. At ring atom C5 the propyl substituent adopts a *trans* conformation, and the corresponding torsion angle C5—C8—C9—C10 is −164.80 (13)°. The C5—C8—C9—C10 fragment is twisted significantly out of the plane defined by atoms C8, C5 and C2, which bis­ects the pyrimidine­trione fragment into two approximately sym­met­rical halves, resulting in a pseudo-torsion angle C2⋯C5—C8—C9 of −125.69 (11)°. Closer inspection suggests that this particular geometry may help to prevent unfavourably close intra­molecular contacts between the O7 hy­droxy group and the CH_2_ group at C9, and may be also facilitate the participation of the hy­droxy group in complex inter­molecular hydrogen-bonding inter­actions.

## Supra­molecular features   

**Figure 2:**
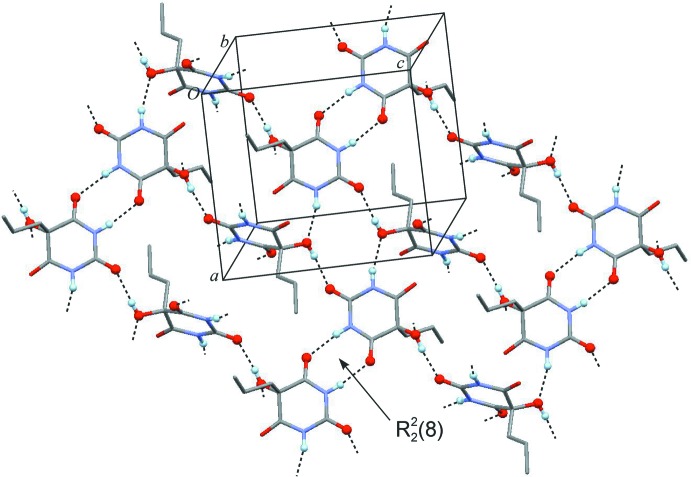
Layer fragment of the H-bonded framework which contains rings connecting four and six mol­ecules in addition to 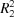
(8) rings. Hydrogen bonds are drawn as dashed lines. H and O atoms engaged in hydrogen bonding are drawn as balls and all the other H atoms are omitted for clarity.

**Figure 3:**
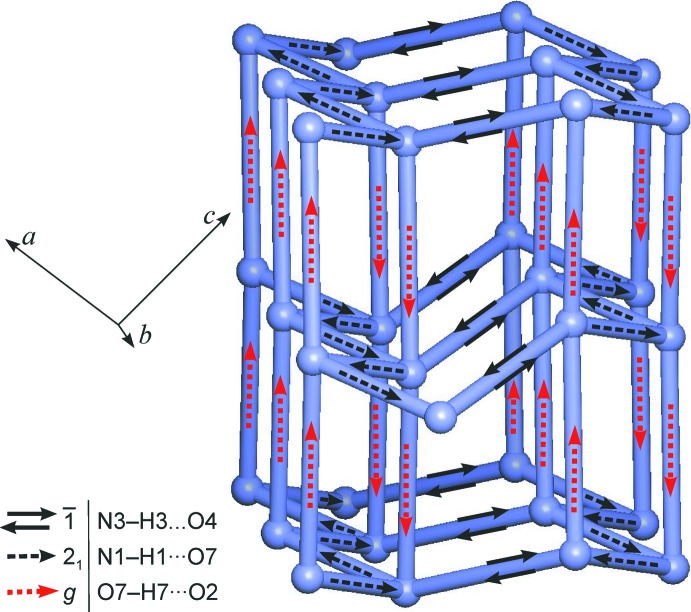
The N—H⋯O(carbonyl), N—H⋯O(hy­droxy) and O—H⋯O(carbonyl) bonded *F*6_5_[4^4^.6^6^-**nov**] structure of title compound. Mol­ecules are represented as nodes and their hydrogen-bond connections as links between them.

One NH group and one carbonyl group of the mol­ecule are engaged in a centrosymmetric two-point inter­action, N3—H3⋯O4^ii^ (Table 1 ▸), resulting in an 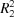
(8) ring (Etter *et al.*, 1990 ▸; Bernstein *et al.*, 1995 ▸). This kind of ring is a ubiquitous feature in crystal structures of barbiturates (Gelbrich *et al.*, 2011 ▸). The other NH group is bonded to the hy­droxy group of a second mol­ecule *via* a 2_1_ operation, N1—H1⋯O7^i^, and this inter­action is accompanied by a short O6⋯C4^i^ contact [2.8654 (18) Å]. Additionally, the hy­droxy group donates a hydrogen bond to the C2 carbonyl group of another mol­ecule related by glide symmetry (O7—H7⋯O2^iii^). Altogether, six hydrogen bonds connect each mol­ecule to five other mol­ecules. In addition to the aforementioned 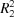
(8) rings, the resulting hydrogen-bonded framework structure also displays rings composed of four and six mol­ecules (Fig. 2 ▸). This 5-connected framework has the topology of the **nov** structure (Blatov *et al.*, 2004 ▸). Fig. 3 ▸ shows a graph of the hydrogen-bonded structure (HBS) according to the methodology proposed by Hursthouse *et al.* (2015 ▸). The short descriptor according to Hursthouse *et al.* (2015 ▸) for this HBS is *F*6_5_[4^4^.6^6^-**nov**].

## Database survey   

The Cambridge Structural Database (Version 5.36; Groom & Allen, 2014 ▸) contains the crystal structure of 5,5-di­hydroxy­barbituric acid (Singh, 1965 ▸; Harrowfield *et al.*, 1989 ▸; CSD refcode ALXANM01) and those of a monohydrate (Lewis & Tocher, 2004 ▸; PAGYUS), a trihydrate (Lewis & Tocher, 2004*b* ▸; HBARBT01) and a 1,4-dioxane hemisolvate (Gelbrich *et al.*, 2010 ▸; NUQYII) of the same compound. Two-point connections based on N—H⋯O=C bonds which result in characteristic 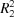
(8) rings are found in each of these compounds.

**Figure 4:**
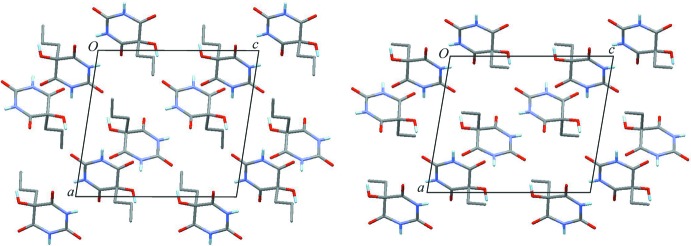
An illustration of the similar packing of mol­ecules in the title compound (left) and its ethyl analogue (right). Each structure is viewed along its [010] direction. H atoms in alkyl groups are omitted for clarity.

The title structure displays just one such inter­action which involves the carbonyl group at ring position 4 (Fig. 4 ▸). One such connection, albeit *via* the C2 carbonyl group, also exists in the 5,5-di­hydroxy­barbituric acid structure. Here it forms part of the *C*-4 ladder motif which is known from 5,5-disubstituted derivatives of barbituric acid (Gelbrich *et al.*, 2011 ▸).

The monohydrate and 1,4-dioxane hemisolvate each contain two two-point N—H⋯O=C connections per mol­ecule, in the first case *via* the topologically equivalent C4 and C6 carbonyl groups and in the second *via* the C4 and C2 carbonyl groups, resulting in the looped chain motifs *C*-2 and *C*-1 (Gelbrich *et al.*, 2011 ▸), respectively, which are frequently encountered in barbiturates. *C*-2 chains are also found in the structure of the trihydrate. The mol­ecular conformation of 5-hy­droxy-5-ethyl­barbituric acid (Gatehouse & Craven, 1971 ▸; HEBARB) is similar to that of the title structure with respect to the pseudo-torsion angle of 124.3°, which is structurally analogous to the C2⋯C5—C8—C9 angle discussed above. A comparison with the program *XPac* (for details, see below) indicated that these two compounds are indeed isostructural. Geometrical differences between the two mol­ecular packing arrangements are small (Fig. 4 ▸), which is reflected in a calculated *XPac* dissimilarity index of just 5.4. This close packing similarity is remarkable insofar as the substitution of a propyl with an ethyl group alters the mol­ecular shape considerably and leads to an 11% decrease in the volume of the unit cell. The unit-cell parameters of the two isostructures correspond directly with one another. The *a* and *b* axes of the ethyl analogue (determined at room temperature) are 6.1% and 6.5% shorter than those of the title compound. Simultaneously, the *c* axis of the ethyl analogue is 1.5% longer and the β angle is enlarged by 1.0°.

## Synthesis and crystallization   

A glass slide with a sample of 5-propyl­barbituric acid embedded in paraffin oil was placed on a hot bench. The sample was melted and left to crystallize. Within a few days, the original crystals had partially converted and cube-shaped single crystals of the title compound had formed.

## Refinement   

Crystal data, data collection and structure refinement details are summarised in Table 2 ▸. The data collection was carried out in the manner described by Coles & Gale (2012 ▸). All H atoms were identified in difference maps. Methyl H atoms were idealized and included as rigid groups allowed to rotate but not tip (C—H = 0.98 Å). H atoms bonded to secondary CH_2_ carbon atoms were positioned geometrically (C—H = 0.99 Å). Hydrogen atoms bonded to N atoms were refined with restrained distances [N—H = 0.86 (1) Å]. The hydrogen atom of the hy­droxy group was refined freely and the *U*_iso_ parameters of all hydrogen atoms were also refined freely.

## Analysis of structural features   

The topology of the HBS was determined and classified with the programs *ADS* and *IsoTest* of the *TOPOS* package (Blatov, 2006 ▸) in the manner described by Baburin & Blatov (2007 ▸). The topology graph for the HBS (Fig. 3 ▸) is based on a net drawn with the *IsoCryst* program of the *TOPOS* package. The HBS of the title structure was defined by the three inter­actions N—H⋯O(carbon­yl), N—H⋯O(hy­droxy) and O—H⋯O(carbon­yl) listed in Table 1 ▸. The mol­ecular packing in the title compound and its ethyl analogue were compared using the program *XPac* (Gelbrich & Hursthouse, 2005 ▸). The underlying calculations were based on a comparison of sets of inter­molecular geometrical parameters generated from all non-H atomic positions of the title compound, except for the methyl carbon atom, and all 12 non-H atomic positions of the ethyl analogue. A match of two complete clusters consisting of a central mol­ecule and 17 coordinating mol­ecules was obtained with a dissimilarity index (Gelbrich *et al.*, 2012 ▸) of 5.4, indicating isostructurality of the two compounds with a high degree of packing similarity.

## Supplementary Material

Crystal structure: contains datablock(s) I, global. DOI: 10.1107/S2056989015018769/zs2347sup1.cif

Structure factors: contains datablock(s) I. DOI: 10.1107/S2056989015018769/zs2347Isup2.hkl

CCDC reference: 1429681

Additional supporting information:  crystallographic information; 3D view; checkCIF report

## Figures and Tables

**Table 1:** Hydrogen-bond geometry (, )

*D*H*A*	*D*H	H*A*	*D* *A*	*D*H*A*
N1H1O7^i^	0.87(1)	2.03(1)	2.8683(17)	164(2)
N3H3O4^ii^	0.86(1)	2.00(1)	2.8451(16)	170(2)
O7H7O2^iii^	0.84(2)	1.98(2)	2.8055(15)	169(2)

Symmetry codes: (i) 
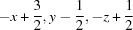
; (ii) 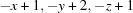; (iii) 
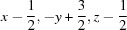
.

**Table 2:** Experimental details

Crystal data	
Chemical formula	C_7_H_10_N_2_O_4_
*M*_r_	186.17
Crystal system, space group	Monoclinic, *P*2_1_/*n*
Temperature (K)	100
*a*, *b*, *c* ()	10.7862(8), 6.7093(5), 11.7365(6)
()	98.632(6)
*V* (^3^)	839.72(10)
*Z*	4
Radiation type	Mo *K*
(mm^1^)	0.12
Crystal size (mm)	0.05 0.05 0.05

Data collection	
Diffractometer	Rigaku Saturn724+
Absorption correction	Multi-scan (*CrysAlis PRO*; Agilent, 2014 ▸)
*T*_min_, *T*_max_	0.809, 1.000
No. of measured, independent and observed [*I* > 2(*I*)] reflections	5354, 1724, 1354
*R*_int_	0.034
(sin /)_max_ (^1^)	0.625

Refinement	
*R*[*F* ^2^ > 2(*F* ^2^)], *wR*(*F* ^2^), *S*	0.037, 0.095, 1.05
No. of reflections	1724
No. of parameters	138
No. of restraints	2
H-atom treatment	H atoms treated by a mixture of independent and constrained refinement
_max_, _min_ (e ^3^)	0.29, 0.20

Computer programs: *CrystalClear-SM Expert* (Rigaku, 2012 ▸), *CrysAlis PRO* (Agilent, 2014 ▸), *SHELXT* (Sheldrick, 2015*a* ▸), *SHELXL2014*/6 (Sheldrick, 2015*b* ▸), *XP* in *SHELXTL* (Sheldrick, 2008 ▸8) and *Mercury* (Macrae *et al.*, 2006 ▸) and *publCIF* (Westrip, 2010 ▸).

## supplementary crystallographic information

### Crystal data

**Table:**

C_7_H_10_N_2_O_4_	*F*(000) = 392
*M**_r_* = 186.17	*D*_x_ = 1.473 Mg m^−^^3^
Monoclinic, *P*2_1_/*n*	Mo *K*α radiation, λ = 0.71073 Å
*a* = 10.7862 (8) Å	Cell parameters from 3013 reflections
*b* = 6.7093 (5) Å	θ = 2.4–27.5°
*c* = 11.7365 (6) Å	µ = 0.12 mm^−^^1^
β = 98.632 (6)°	*T* = 100 K
*V* = 839.72 (10) Å^3^	Cube, colourless
*Z* = 4	0.05 × 0.05 × 0.05 mm

### Data collection

**Table:**

Rigaku Saturn724+ diffractometer	1724 independent reflections
Radiation source: Sealed Tube	1354 reflections with *I* > 2σ(*I*)
Graphite Monochromator monochromator	*R*_int_ = 0.034
Detector resolution: 28.5714 pixels mm^-1^	θ_max_ = 26.4°, θ_min_ = 2.4°
profile data from ω–scans	*h* = −12→13
Absorption correction: multi-scan (*CrysAlis PRO*; Agilent, 2014)	*k* = −8→8
*T*_min_ = 0.809, *T*_max_ = 1.000	*l* = −14→14
5354 measured reflections	

### Refinement

**Table:**

Refinement on *F*^2^	Primary atom site location: structure-invariant direct methods
Least-squares matrix: full	Secondary atom site location: difference Fourier map
*R*[*F*^2^ > 2σ(*F*^2^)] = 0.037	Hydrogen site location: mixed
*wR*(*F*^2^) = 0.095	H atoms treated by a mixture of independent and constrained refinement
*S* = 1.05	*w* = 1/[σ^2^(*F*_o_^2^) + (0.0498*P*)^2^ + 0.1441*P*] where *P* = (*F*_o_^2^ + 2*F*_c_^2^)/3
1724 reflections	(Δ/σ)_max_ < 0.001
138 parameters	Δρ_max_ = 0.29 e Å^−^^3^
2 restraints	Δρ_min_ = −0.20 e Å^−^^3^

### Special details

**Table:**

Geometry. All e.s.d.'s (except the e.s.d. in the dihedral angle between two l.s. planes) are estimated using the full covariance matrix. The cell e.s.d.'s are taken into account individually in the estimation of e.s.d.'s in distances, angles and torsion angles; correlations between e.s.d.'s in cell parameters are only used when they are defined by crystal symmetry. An approximate (isotropic) treatment of cell e.s.d.'s is used for estimating e.s.d.'s involving l.s. planes.

### Fractional atomic coordinates and isotropic or equivalent isotropic displacement parameters (Å^2^)

**Table:**

	*x*	*y*	*z*	*U*_iso_*/*U*_eq_	
N1	0.75066 (12)	0.60615 (19)	0.37252 (9)	0.0154 (3)	
H1	0.8189 (12)	0.536 (3)	0.3792 (16)	0.033 (5)*	
O2	0.78456 (10)	0.66277 (16)	0.56534 (8)	0.0201 (3)	
C2	0.72112 (13)	0.6895 (2)	0.47207 (11)	0.0147 (3)	
N3	0.61584 (11)	0.80699 (19)	0.46014 (9)	0.0150 (3)	
H3	0.5995 (14)	0.870 (2)	0.5200 (10)	0.020 (4)*	
O4	0.47047 (9)	0.99725 (16)	0.35153 (8)	0.0180 (3)	
C4	0.54700 (13)	0.8640 (2)	0.35743 (11)	0.0139 (3)	
C5	0.56139 (13)	0.7360 (2)	0.25307 (11)	0.0140 (3)	
O6	0.73523 (10)	0.58567 (18)	0.17883 (8)	0.0220 (3)	
C6	0.68995 (13)	0.6406 (2)	0.26184 (11)	0.0153 (3)	
O7	0.54144 (10)	0.85117 (16)	0.15172 (8)	0.0161 (3)	
H7	0.465 (2)	0.864 (3)	0.1270 (16)	0.040 (6)*	
C8	0.46287 (14)	0.5674 (2)	0.25253 (11)	0.0158 (3)	
H8A	0.4849	0.4858	0.3229	0.021 (4)*	
H8B	0.3799	0.6284	0.2557	0.016 (4)*	
C9	0.45248 (15)	0.4312 (2)	0.14778 (13)	0.0225 (4)	
H9A	0.4526	0.5128	0.0775	0.035 (5)*	
H9B	0.5262	0.3417	0.1553	0.033 (5)*	
C10	0.33380 (15)	0.3070 (3)	0.13545 (13)	0.0247 (4)	
H10A	0.3383	0.2139	0.2003	0.034 (5)*	
H10B	0.3253	0.2319	0.0631	0.036 (5)*	
H10C	0.2611	0.3948	0.1350	0.044 (6)*	

### Atomic displacement parameters (Å^2^)

**Table:**

	*U*^11^	*U*^22^	*U*^33^	*U*^12^	*U*^13^	*U*^23^
N1	0.0131 (7)	0.0172 (7)	0.0154 (6)	0.0033 (6)	0.0002 (5)	−0.0013 (5)
O2	0.0199 (6)	0.0225 (6)	0.0159 (5)	0.0037 (5)	−0.0043 (4)	−0.0010 (4)
C2	0.0132 (8)	0.0136 (8)	0.0168 (7)	−0.0009 (6)	0.0004 (5)	0.0002 (5)
N3	0.0160 (7)	0.0173 (7)	0.0114 (6)	0.0039 (5)	0.0011 (5)	−0.0022 (5)
O4	0.0170 (6)	0.0205 (6)	0.0161 (5)	0.0058 (5)	0.0005 (4)	−0.0001 (4)
C4	0.0110 (8)	0.0155 (8)	0.0151 (7)	−0.0018 (6)	0.0016 (5)	0.0005 (5)
C5	0.0127 (8)	0.0169 (8)	0.0117 (6)	0.0010 (6)	−0.0003 (5)	0.0009 (5)
O6	0.0173 (6)	0.0323 (7)	0.0168 (5)	0.0046 (5)	0.0042 (4)	−0.0025 (5)
C6	0.0143 (8)	0.0161 (8)	0.0152 (7)	−0.0040 (6)	0.0009 (5)	−0.0003 (5)
O7	0.0143 (6)	0.0202 (6)	0.0128 (5)	−0.0006 (5)	−0.0009 (4)	0.0031 (4)
C8	0.0134 (8)	0.0178 (8)	0.0161 (6)	−0.0004 (6)	0.0021 (5)	0.0014 (6)
C9	0.0226 (9)	0.0200 (9)	0.0256 (8)	−0.0019 (7)	0.0061 (6)	−0.0065 (7)
C10	0.0262 (10)	0.0202 (9)	0.0264 (8)	−0.0030 (7)	−0.0002 (6)	0.0003 (7)

### Geometric parameters (Å, º)

**Table:**

N1—C2	1.3754 (18)	O6—C6	1.2110 (17)
N1—C6	1.3836 (16)	O7—H7	0.84 (2)
N1—H1	0.866 (9)	C8—C9	1.5226 (19)
O2—C2	1.2141 (16)	C8—H8A	0.9900
C2—N3	1.3720 (19)	C8—H8B	0.9900
N3—C4	1.3719 (17)	C9—C10	1.516 (2)
N3—H3	0.860 (9)	C9—H9A	0.9900
O4—C4	1.2117 (17)	C9—H9B	0.9900
C4—C5	1.5228 (19)	C10—H10A	0.9800
C5—O7	1.4076 (16)	C10—H10B	0.9800
C5—C6	1.517 (2)	C10—H10C	0.9800
C5—C8	1.551 (2)		
			
C2—N1—C6	126.31 (13)	N1—C6—C5	115.68 (12)
C2—N1—H1	116.4 (12)	C5—O7—H7	111.6 (14)
C6—N1—H1	116.8 (12)	C9—C8—C5	114.10 (12)
O2—C2—N3	121.52 (13)	C9—C8—H8A	108.7
O2—C2—N1	122.33 (14)	C5—C8—H8A	108.7
N3—C2—N1	116.15 (11)	C9—C8—H8B	108.7
C2—N3—C4	125.47 (12)	C5—C8—H8B	108.7
C2—N3—H3	117.8 (10)	H8A—C8—H8B	107.6
C4—N3—H3	115.6 (11)	C10—C9—C8	111.45 (13)
O4—C4—N3	122.02 (12)	C10—C9—H9A	109.3
O4—C4—C5	121.39 (11)	C8—C9—H9A	109.3
N3—C4—C5	116.26 (13)	C10—C9—H9B	109.3
O7—C5—C6	108.13 (11)	C8—C9—H9B	109.3
O7—C5—C4	110.43 (12)	H9A—C9—H9B	108.0
C6—C5—C4	112.70 (11)	C9—C10—H10A	109.5
O7—C5—C8	112.27 (11)	C9—C10—H10B	109.5
C6—C5—C8	108.15 (12)	H10A—C10—H10B	109.5
C4—C5—C8	105.19 (11)	C9—C10—H10C	109.5
O6—C6—N1	120.87 (14)	H10A—C10—H10C	109.5
O6—C6—C5	123.30 (12)	H10B—C10—H10C	109.5
			
C6—N1—C2—O2	−174.59 (14)	C2—N1—C6—C5	−16.4 (2)
C6—N1—C2—N3	4.9 (2)	O7—C5—C6—O6	−34.5 (2)
O2—C2—N3—C4	172.08 (14)	C4—C5—C6—O6	−156.87 (14)
N1—C2—N3—C4	−7.4 (2)	C8—C5—C6—O6	87.29 (17)
C2—N3—C4—O4	−165.40 (14)	O7—C5—C6—N1	149.92 (12)
C2—N3—C4—C5	21.1 (2)	C4—C5—C6—N1	27.56 (18)
O4—C4—C5—O7	35.46 (19)	C8—C5—C6—N1	−88.28 (15)
N3—C4—C5—O7	−151.01 (12)	O7—C5—C8—C9	54.47 (16)
O4—C4—C5—C6	156.51 (14)	C6—C5—C8—C9	−64.75 (14)
N3—C4—C5—C6	−29.96 (18)	C4—C5—C8—C9	174.60 (12)
O4—C4—C5—C8	−85.88 (16)	C5—C8—C9—C10	−164.80 (13)
N3—C4—C5—C8	87.64 (14)	C2—C5—C8—C9	−125.69 (11)
C2—N1—C6—O6	167.92 (14)		

### Hydrogen-bond geometry (Å, º)

**Table:**

*D*—H···*A*	*D*—H	H···*A*	*D*···*A*	*D*—H···*A*
N1—H1···O7^i^	0.87 (1)	2.03 (1)	2.8683 (17)	164 (2)
N3—H3···O4^ii^	0.86 (1)	2.00 (1)	2.8451 (16)	170 (2)
O7—H7···O2^iii^	0.84 (2)	1.98 (2)	2.8055 (15)	169 (2)

Symmetry codes: (i) −*x*+3/2, *y*−1/2, −*z*+1/2; (ii) −*x*+1, −*y*+2, −*z*+1; (iii) *x*−1/2, −*y*+3/2, *z*−1/2.
